# Association of Pretreatment Immune-Inflammatory Biomarkers with Pathological Tumor Regression Following Neoadjuvant Chemoradiotherapy in Locally Advanced Rectal Cancer

**DOI:** 10.3390/jcm15135039

**Published:** 2026-06-28

**Authors:** Ahmet Sencer Ergin, Burcin Cakan Demirel, Sahin Bedir, Nida Sünnetci Arıkan, Alparslan Saylar, Ali Karabulut, Nihat Bugdayci, Tevhide Bilgen Özcan, Hüsniye Esra Pasaoglu

**Affiliations:** 1 Department of General Surgery, Bagcilar Training and Research Hospital, University of Health Sciences, 34200 Istanbul, Turkey; saylaralparslan@gmail.com (A.S.); alikarabulut76767@gmail.com (A.K.); nihatbugdayci@gmail.com (N.B.); 2 Department of Medical Oncology, Medipol Mega University Hospital, Istanbul Medipol University, 34124 Istanbul, Turkey; burcin.cakandemirel@gmail.com; 3 Department of Medical Oncology, Faculty of Medicine, Gaziosmanpaşa Hospital, Istinye University, 34250 Istanbul, Turkey; sahinbedir7786@hotmail.com; 4 Department of Radiation Oncology, Bagcilar Training and Research Hospital, University of Health Sciences, 34164 Istanbul, Turkey; nidasun@gmail.com; 5 Department of Pathology, Bagcilar Training and Research Hospital, University of Health Sciences, 34200 Istanbul, Turkey; tevhidebilgen@yahoo.com.tr (T.B.Ö.); esrapasa2310@gmail.com (H.E.P.)

**Keywords:** rectal cancer, neoadjuvant chemoradiotherapy, tumor regression grade, pathological complete response, carcinoembryonic antigen, inflammation biomarkers

## Abstract

**Background:** Predicting tumor regression following neoadjuvant chemoradiotherapy (nCRT) remains a major challenge in the management of locally advanced rectal cancer (LARC). Readily available inflammatory biomarkers may provide useful information regarding treatment response. **Methods:** This retrospective single-center study included 88 patients with stage II–III rectal adenocarcinoma who underwent neoadjuvant chemoradiotherapy followed by curative-intent surgery between 2017 and 2025. Patients were classified according to the College of American Pathologists tumor regression grading system as CAP 0 (pathological complete response, *n* = 22), CAP 2 (partial response, *n* = 50), or CAP 3 (poor/no response, *n* = 16). Pretreatment C-reactive protein, carcinoembryonic antigen (CEA), neutrophil, platelet, monocyte, and lymphocyte counts, together with NLR, PLR, and PIV, were compared across groups. Receiver operating characteristic and logistic regression analyses were performed for pathological complete response (pCR). **Results:** None of the evaluated biomarkers differed significantly across CAP groups. The smallest omnibus *p*-values were observed for neutrophil count (*p* = 0.052), monocyte count (*p* = 0.075), and CEA (*p* = 0.088). Monocyte count showed the highest discriminatory performance for pathological complete response (AUC = 0.663), followed by CEA (AUC = 0.640). In sensitivity analyses adjusted for baseline clinical T stage and receipt of total neoadjuvant therapy, CEA, neutrophil count, and monocyte count were not independently associated with pathological complete response. More favorable tumor regression was associated with lower residual tumor burden and reduced nodal involvement. **Conclusions:** Pretreatment inflammatory biomarkers showed biologically plausible numerical patterns across tumor regression groups, but their discriminatory and independent predictive performance was limited. These markers should not be considered stand-alone clinical prediction tools and should be validated within larger, multimodal prospective models.

## 1. Introduction

Colorectal cancer is the third most commonly diagnosed malignancy and the second leading cause of cancer death worldwide, with an estimated 1.9 million new cases recorded in 2022 [1]. Rectal cancers account for roughly one-third of this burden and pose distinct therapeutic challenges, owing to anatomical proximity to the sphincter complex, pelvic sidewall vasculature, and surrounding visceral structures.

The management of locally advanced rectal cancer (LARC; clinical stage II–III) has shifted substantially over the past two decades. For many years, the standard of care was neoadjuvant long-course chemoradiotherapy (CRT) or short-course radiotherapy (SCRT) followed by total mesorectal excision (TME) and, in selected cases, adjuvant chemotherapy [2,3,4].

One of the most clinically relevant endpoints of nCRT is pathological complete response (pCR), defined as the eradication of viable tumor cells from both the primary site and regional lymph nodes (ypT0N0). pCR is associated with favorable long-term outcomes in retrospective series and pooled analyses [5,6]. Nevertheless, pCR occurs in only 15–30% of patients treated with standard long-course nCRT; most patients have a partial response, while a smaller proportion show minimal or no histological regression [7].

To improve systemic disease control and treatment completion, total neoadjuvant therapy (TNT)—the delivery of systemic chemotherapy before surgery in addition to radiotherapy—has become a standard option for many patients with intermediate- to high-risk LARC. Randomized trials have demonstrated improved disease control and higher response rates with TNT, expanding opportunities for organ-preservation strategies in carefully selected patients [8,9,10,11]. For the molecularly distinct subgroup of mismatch repair-deficient (dMMR/MSI-H) rectal cancers, neoadjuvant immunotherapy has shown remarkable efficacy, highlighting the growing importance of individualized treatment approaches [12]. Despite these advances, substantial heterogeneity in treatment response persists, underscoring the need for readily available biomarkers capable of predicting tumor regression and identifying patients with distinct patterns of tumor response to neoadjuvant treatment.

The systemic inflammatory response has emerged as a key determinant of tumor biology and treatment sensitivity. Chronic inflammation promotes tumor progression, facilitates immune evasion, and may directly attenuate the cytotoxic effects of chemoradiotherapy through hypoxia-induced pathways, DNA repair upregulation, and the expansion of immunosuppressive cell populations within the tumor microenvironment [13]. Among the most accessible inflammatory biomarkers in routine clinical practice are C-reactive protein (CRP), carcinoembryonic antigen (CEA), and the cellular components of the peripheral blood count: absolute neutrophil, platelet, monocyte, and lymphocyte counts. These six parameters, collectively termed pan-immune-inflammatory markers in the present study, reflect distinct but overlapping aspects of systemic immune activation—from acute-phase reactant production and tumor antigen shedding to granulocyte mobilization, platelet-mediated metastatic facilitation, monocyte-to-macrophage polarization, and adaptive lymphocyte-driven antitumor immunity [14,15].

Several composite indices derived from these individual values—most notably the neutrophil-to-lymphocyte ratio (NLR) and platelet-to-lymphocyte ratio (PLR)—have been studied extensively in colorectal cancer, with consistent associations reported between elevated NLR and PLR and poorer oncologic outcomes [16,17]. However, the specific relationship between individual pretreatment inflammatory cell counts and post-nCRT tumor regression grade—as formally defined by the College of American Pathologists (CAP) system—has not been comprehensively evaluated in the Turkish LARC population, and published series from this region remain scarce.

The present study, therefore, aimed to determine whether pretreatment CRP, CEA, neutrophil, platelet, monocyte, and lymphocyte counts differ across patients stratified by the CAP tumor regression score, and to characterize the associated pre- and posttreatment clinicopathological landscape—including T- and N-stage transition patterns and lymph node burden—across response groups in a single-center cohort of 88 consecutive patients treated between 2017 and 2025.

## 2. Materials and Methods

This retrospective single-center cohort study was conducted at a tertiary academic referral hospital. Consecutive patients with histologically confirmed primary rectal adenocarcinoma who underwent long-course neoadjuvant chemoradiotherapy (nCRT), followed by curative-intent surgical resection between June 2017 and January 2025, were screened for eligibility. Institutional pathology archives and operative registries from the Department of General Surgery were systematically cross-referenced to identify the study population.

### 2.1. Inclusion Criteria

Patients were eligible if they met all of the following criteria:

1. Clinical stage II–III disease (cT3–4 and/or cN1–2), determined by pelvic magnetic resonance imaging (MRI);
2. Age above 18 years;
3. Histopathological diagnosis of adenocarcinoma;
4. Rectal cancer as the sole primary malignancy;
5. Completion of long-course nCRT, followed by radical surgical resection with curative intent.

### 2.2. Exclusion Criteria

Patients were excluded if they:

1. Received systemic corticosteroids or immunosuppressive therapy for concomitant medical conditions;
2. Had incomplete clinicopathological or laboratory data precluding statistical analysis;
3. Underwent surgical treatment at an external institution;
4. Presented with metastatic disease at the time of diagnosis.

### 2.3. Neoadjuvant Chemoradiotherapy Protocol

Neoadjuvant treatment consisted of long-course chemoradiotherapy with concurrent fluoropyrimidine-based chemotherapy, followed by curative-intent total mesorectal excision. Depending on the treatment period and multidisciplinary team decision, selected patients received induction FOLFOX (folinic acid, fluorouracil, and oxaliplatin) or CAPOX (capecitabine and oxaliplatin) before chemoradiotherapy as a part of TNT. Use of TNT increased during the later study years, while the long-course chemoradiotherapy and surgical backbone remained consistent. Overall, 49 patients (55.7%) received TNT and 39 (44.3%) received conventional long-course chemoradiotherapy without induction chemotherapy.

### 2.4. Pathological Evaluation and CAP Regression Grading

All resection specimens were evaluated by dedicated gastrointestinal pathologists at the institutional pathology department following standard histopathological processing protocols. Tumor regression following neoadjuvant chemoradiotherapy was assessed using the College of American Pathologists (CAP) tumor regression grading (TRG) system. According to this classification:

- A CAP Score of 0 indicates a complete response with no viable residual tumor cells;
- A CAP Score of 1 indicates near-complete response with only single cells or rare small groups of tumor cells;
- A CAP Score of 2 indicates a partial response with evident residual tumor accompanied by treatment-related fibrosis;
- A CAP Score of 3 indicates poor or no response with extensive residual tumor and minimal or absent regression changes.

No patients were classified as CAP Grade 1 in the present cohort. Therefore, the intermediate-response group consisted exclusively of patients with CAP Grade 2 tumors and is referred to as the partial response group throughout the manuscript.

Final study groups, therefore, consisted of:

- Score 3: no response;
- Score 2: partial response;
- Score 0: pathological complete response (pCR).

Posttreatment pathological staging was recorded according to the 8th edition of the American Joint Committee on Cancer (AJCC) TNM classification system.

### 2.5. Pretreatment Laboratory Variables

All measurements were obtained from routine pretreatment blood work collected within four weeks prior to nCRT initiation. The following six parameters were analyzed: CRP (mg/L), CEA (ng/mL), absolute neutrophil count (×10^3^/mm^3^), platelet count (×10^3^/mm^3^), absolute monocyte count (×10^3^/mm^3^), and absolute lymphocyte count (×10^3^/mm^3^). Derived inflammatory indices were calculated from the same pretreatment complete blood count as follows: the neutrophil-to-lymphocyte ratio (NLR) was calculated by dividing the absolute neutrophil count by the absolute lymphocyte count; the platelet-to-lymphocyte ratio (PLR) was calculated by dividing the platelet count by the absolute lymphocyte count; and the pan-immune-inflammation value (PIV) was calculated as (absolute neutrophil count × platelet count × absolute monocyte count)/absolute lymphocyte count. All blood cell counts were entered in ×10^3^/mm^3^.

All laboratory analyses were performed in the same institutional central laboratory using standardized quality-control procedures throughout the study period. No major changes in analytical methodology that would be expected to affect the reported parameters occurred during the study period.

### 2.6. Statistical Analysis

Statistical analyses were performed using Python 3.11 with the SciPy and Statsmodels libraries in a Jupyter Notebook environment.

Normality of continuous variables was assessed using the Shapiro–Wilk test. Because most variables deviated from a normal distribution, continuous variables were compared across CAP groups using the Kruskal–Wallis H test. Categorical variables were compared using the chi-square test or Fisher’s exact test, as appropriate. ROC curve analysis was used to evaluate the ability of each pretreatment biomarker to discriminate pCR, defined as ypT0N0 (CAP Score 0), from non-pCR (CAP Scores 2 and 3). The optimal cut-off value was determined using the Youden index (sensitivity + specificity − 1). For biomarkers inversely related to pCR, lower values were defined as test-positive so that the reported AUC reflected discrimination in the hypothesized direction. AUCs were reported with 95% confidence intervals obtained by bootstrap resampling with 2000 iterations; sensitivity and specificity were calculated at the optimal cut-off.

Univariable logistic regression analyses were performed for each biomarker, with odds ratios reported per one-standard-deviation increase to facilitate comparison. Continuous variables are presented as mean ± standard deviation or median [interquartile range], and categorical variables as a number (percentage). All tests were two-sided, and *p* < 0.05 was considered statistically significant. Given the limited number of pCR events, regression analyses were considered exploratory.

To assess potential confounding by baseline clinical T stage and treatment regimen, three separate multivariable logistic regression sensitivity models were constructed. Each model included one standardized candidate biomarker—CEA, neutrophil count, or monocyte count—together with baseline cT category (cT4 versus cT2–3) and receipt of TNT (yes versus no). Adjusted odds ratios were reported per one-standard-deviation increase with 95% confidence intervals and two-sided *p*-values. Exploratory treatment-stratified analyses were also performed separately in patients who received TNT and those who received conventional long-course chemoradiotherapy; within each treatment subgroup, biomarker distributions across CAP groups were compared using Kruskal–Wallis tests. Because of the small subgroup sizes and multiple comparisons, these subgroup analyses were considered descriptive, and no multiplicity-adjusted inference was made.

The authors used ChatGPT version 5.4 (OpenAI) for language editing and grammatical revision of the manuscript. All scientific content, data analysis, interpretations, and conclusions were developed and verified by the authors.

## 3. Results

### 3.1. Patient Demographics and Baseline Clinical Characteristics

A total of 88 patients with locally advanced rectal adenocarcinoma who underwent neoadjuvant chemotherapy and long-course neoadjuvant chemoradiotherapy (nCRT), followed by curative-intent surgery, were included in the study. According to the College of American Pathologists (CAP) tumor regression grading system, 22 patients (25.0%) achieved a pathological complete response (CAP Score 0), 50 patients (56.8%) demonstrated a partial response (CAP Score 2), and 16 patients (18.2%) showed poor or absent tumor regression (CAP Score 3).

The baseline demographic and clinicopathological characteristics are summarized in Table 1. No statistically significant differences were observed among the CAP response groups regarding age, body mass index (BMI), sex distribution, American Society of Anesthesiologists (ASA) score, or length of hospital stay (all *p* > 0.05). Tumor distance from the anal verge differed across the response groups in the omnibus analysis (*p* = 0.040).

Baseline cT stage showed a borderline difference across the CAP response groups, with cT4 disease present in 54.5% of patients with CAP 0, 36.0% with CAP 2, and 68.8% with CAP 3 (*p* = 0.051). The proportion receiving total neoadjuvant therapy was comparable across the groups (54.5%, 54.0%, and 62.5%, respectively; *p* = 0.831).

### 3.2. Pretreatment Pan-Immune-Inflammatory Markers

**Figure 1:**
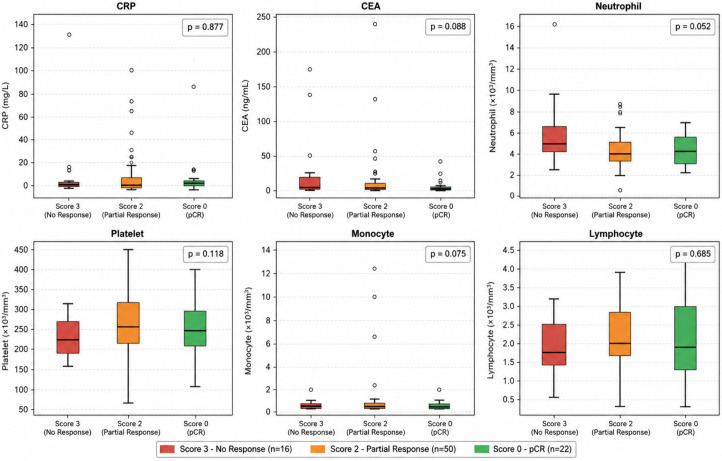
Distribution of pretreatment CRP, CEA, neutrophil, platelet, monocyte, and lymphocyte values across CAP tumor regression groups. Box plots show medians and interquartile ranges; individual points represent patient-level observations. Omnibus Kruskal–Wallis *p*-values are shown in each panel.

The pretreatment inflammatory biomarkers, stratified according to CAP tumor regression groups, are presented in Table 2 and Figure 1. Continuous variables were analyzed using nonparametric methods and are reported as the median [interquartile range].

Among the evaluated biomarkers, pretreatment neutrophil count demonstrated the strongest association trend with pathological response status.

The median neutrophil counts were 4.96 [4.28–6.59], 4.04 [3.37–5.13], and 4.28 [3.25–5.69] ×10^3^/mm^3^ in the CAP 3, CAP 2, and CAP 0 groups, respectively. Although the highest median value was observed in the CAP 3 group, the overall difference did not reach statistical significance (*p* = 0.052).

Similarly, serum carcinoembryonic antigen (CEA) levels showed a progressive decrease across favorable response groups. Patients with a pathological complete response demonstrated lower baseline CEA levels compared with poor responders (2.23 [1.56–4.63] vs. 4.53 [2.36–18.64] ng/mL; *p* = 0.088).

Monocyte count also demonstrated a biologically coherent trend toward association with tumor regression status. Median monocyte levels were lower in the pathological complete response group compared with CAP Score 3 patients (0.44 [0.32–0.56] vs. 0.55 [0.46–0.65] ×10^3^/mm^3^; *p* = 0.075).

No statistically significant differences were observed for CRP, lymphocyte count, platelet count, NLR, PLR, or PIV across the CAP regression groups (all *p* > 0.05).

### 3.3. Pathological Response and Downstaging Findings

Detailed posttreatment pathological staging and lymph-node parameters according to the CAP tumor regression group are presented in Supplementary Tables S1 and S2.

The posttreatment ypT and ypN distributions differed across CAP groups (both *p* < 0.001), reflecting the extent of residual primary and nodal disease captured by pathological regression assessment. All CAP 0 patients fulfilled the ypT0N0 definition of pCR, whereas residual tumor and nodal disease were more frequent in CAP 2 and CAP 3. The pretreatment clinical nodal-stage distribution was comparable among groups (*p* = 0.461).

### 3.4. ROC and Logistic Regression Analyses for Prediction of Pathological Complete Response

**Figure 2:**
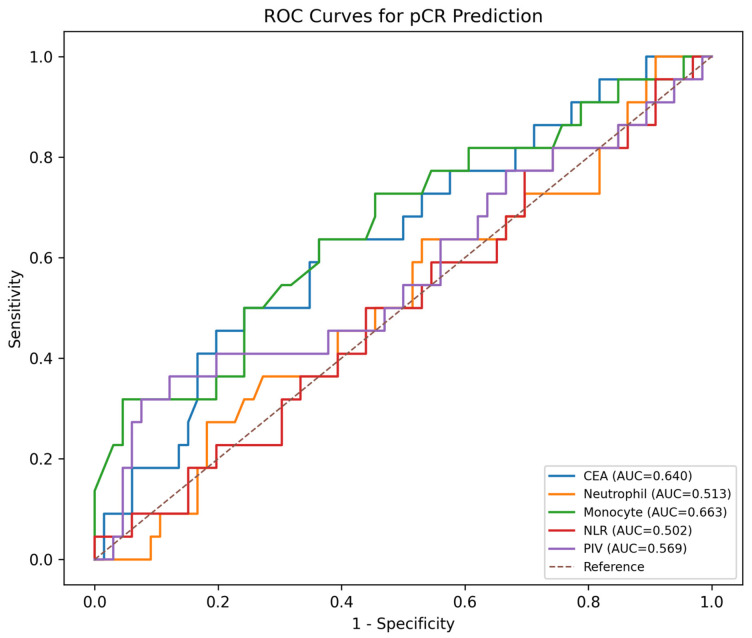
ROC curves for selected pretreatment biomarkers in the discrimination of pathological complete response.

Receiver operating characteristic (ROC) curve analyses were performed to evaluate the discriminatory performance of pretreatment inflammatory biomarkers for predicting a pathological complete response (pCR) following neoadjuvant chemoradiotherapy (Table 3 and Figure 2). Among the evaluated biomarkers, monocyte count demonstrated the highest discriminatory performance with an area under the curve (AUC) of 0.663, followed by CEA (AUC = 0.640). Lower monocyte and CEA levels were associated with an increased likelihood of pCR. In contrast, composite inflammatory indices, including NLR (AUC = 0.502), PLR (AUC = 0.521), and PIV (AUC = 0.569), demonstrated limited predictive utility in this cohort.

Univariate logistic regression analyses, standardized per one-standard-deviation increase, were subsequently performed to assess the association between inflammatory biomarkers and pCR. Monocyte count demonstrated the strongest association trend with a pathological complete response (OR 0.561, 95% CI 0.290–1.083, *p* = 0.085), indicating that lower monocyte levels were associated with a higher probability of achieving pCR. Although CEA also demonstrated a biologically coherent inverse association with pCR (OR 0.381, 95% CI 0.072–2.020, *p* = 0.257), none of the investigated biomarkers reached conventional statistical significance in regression analysis. Overall, these findings suggest modest discriminatory potential for selected inflammatory markers, while robust independent predictive performance was not identified in the present cohort.

CEA, monocyte count, and neutrophil count showed the most relevant discriminatory trends for pCR prediction, whereas composite inflammatory indices, including NLR, PLR, and PIV, demonstrated limited predictive performance in this cohort.

To account for potential confounding by baseline clinical T stage and treatment regimen, additional covariate-adjusted sensitivity analyses were performed. After adjustment for a baseline cT category and receipt of total neoadjuvant therapy, neither CEA (adjusted OR 0.357, 95% CI 0.066–1.932; *p* = 0.232), neutrophil count (adjusted OR 0.899, 95% CI 0.541–1.494; *p* = 0.681), nor monocyte count (adjusted OR 0.547, 95% CI 0.276–1.082; *p* = 0.083) demonstrated an independent association with a pathological complete response. The complete results of these exploratory models are presented in Supplementary Table S3.

Exploratory treatment-stratified analyses did not identify a consistent biomarker pattern. In the TNT subgroup, neutrophil count showed a nominal difference across CAP groups (*p* = 0.010), whereas no other biomarker reached nominal significance; no biomarker differed across CAP groups in the conventional nCRT subgroup. These findings were considered descriptive because of small subgroup sizes and multiple comparisons (Supplementary Table S4).

## 4. Discussion

In this retrospective cohort of 88 patients with locally advanced rectal cancer, we examined the relationship between pretreatment pan-immune-inflammatory biomarkers and tumor regression following neoadjuvant chemoradiotherapy. Several biomarkers exhibited biologically plausible differences among CAP tumor regression groups. Pretreatment neutrophil count showed the strongest association trend with pathological response status, with higher values observed in patients exhibiting little or no tumor regression. Similarly, baseline CEA levels decreased across increasingly favorable response categories, whereas monocyte counts were numerically lower in the CAP 0 group than in the CAP 2 and CAP 3 groups. However, none of the evaluated biomarkers reached conventional statistical significance in regression analyses, and their overall ability to predict pCR remained limited. Additionally, better tumor regression was generally associated with lower residual tumor burden, higher rates of ypN0 status, and more pronounced pathological downstaging. Overall, these findings suggest that readily available inflammatory biomarkers may reflect underlying response biology, although their independent predictive utility appears limited in this cohort.

An important finding of this study is that none of the evaluated biomarkers showed strong independent or clinically meaningful predictive performance despite several biologically plausible trends. This negative result probably reflects the multifactorial nature of tumor response to neoadjuvant therapy, which is affected by tumor burden, molecular features, local immune environment, hypoxia, treatment intensity, and host-related factors that cannot be fully captured by a single peripheral blood parameter. Variations from previous studies may also stem from methodological differences, including patient selection, biomarker cut-off thresholds, timing of blood tests, treatment protocols, and response criteria. Additionally, many published studies mainly focused on survival outcomes or categorized inflammatory indices, whereas this analysis examined pathological regression across CAP response categories. Consequently, the absence of significant findings should not be taken as evidence that systemic inflammation is biologically irrelevant, but rather that these markers have limited utility as sole predictors of pathological response.

### 4.1. The Role of Monocytes and Tumor-Associated Macrophages

Monocyte count was numerically lower in patients with pCR, but neither the overall group comparison nor the regression analyses reached statistical significance. Circulating monocytes can differentiate into tumor-associated macrophages, which may promote tumor progression, suppress antitumor immunity, and contribute to treatment resistance [18]. Previous studies have reported associations between monocyte-related indices and outcomes after nCRT [19,20]. The present finding should therefore be regarded as hypothesis-generating and requires validation in larger cohorts. 

### 4.2. CEA as a Marker of Tumor Regression

Pretreatment CEA values decreased numerically across increasingly favorable CAP groups, with the lowest median observed in patients with pCR. This pattern is consistent with the established role of CEA as a surrogate of tumor burden and with reports of an inverse relationship between baseline CEA and response to nCRT [21,22]. However, the group comparison and logistic regression were not statistically significant, and the wide confidence interval indicates substantial uncertainty.

### 4.3. Neutrophils, NLR, and Other Inflammatory Markers

Pretreatment neutrophil count had the smallest omnibus *p*-value across CAP groups and was highest in CAP 3, but the difference was not statistically significant. Neutrophils may promote tumor progression, angiogenesis, and immune evasion [15], and elevated NLR has been linked to lower pCR rates in previous studies [23].

In contrast, platelet count, lymphocyte count, and derived inflammatory indices, including NLR, PLR, and PIV, did not differ significantly across CAP regression groups. These findings suggest that although systemic inflammatory markers may reflect aspects of tumor biology, their individual predictive utility remains limited. It is possible that more complex measures of the tumor immune microenvironment, such as tumor-infiltrating lymphocytes, offer a more accurate representation of treatment sensitivity than peripheral blood parameters alone [24].

The absence of significant associations for NLR and PLR contrasts with several previous studies [19,25]. Possible explanations include the modest sample size, the small poor-response subgroup, differences in endpoints and statistical approaches, heterogeneity in baseline stage and treatment, and reliance on a single pretreatment measurement. Ratio-based indices may also be unstable when both the numerator and denominator vary. Exploratory treatment-stratified analyses likewise showed no consistent pattern, reinforcing the need for larger studies designed to evaluate treatment-specific effects.

### 4.4. Tumor Downstaging and Lymph Node Findings

Posttreatment residual primary tumor and nodal burden differed across CAP groups, as expected from the regression categories. CAP 3 patients more frequently had residual ypT3–4 and nodal disease, whereas all CAP 0 patients met the ypT0N0 definition of pCR. The lower lymph-node yield observed in better-response groups may reflect treatment-related fibrosis and nodal involution rather than inadequate surgery or pathological assessment [26].

### 4.5. Clinical Implications and Future Directions

The identification of patients who are less likely to achieve favorable tumor regression following standard neoadjuvant chemoradiotherapy remains an important clinical challenge. Total neoadjuvant therapy (TNT) has been shown to improve pathological complete response rates and disease control in several randomized trials [9,10]. Consequently, there is growing interest in developing tools that may help refine treatment selection before therapy initiation.

The best-performing biomarker, monocyte count, achieved an AUC of 0.663, indicating only modest discrimination. Accordingly, these variables are unlikely to be clinically useful as stand-alone predictors. Future studies should evaluate whether they add incremental value to models incorporating MRI features, pathological variables, and molecular biomarkers rather than assessing them in isolation [27,28,29].

### 4.6. Limitations

Several limitations should be considered. First, the retrospective single-center design introduces potential selection bias and limits generalizability. Second, the overall sample size, the small CAP 3 subgroup (*n* = 16), and the limited number of pCR events reduced precision and constrained multivariable model complexity. Multiple biomarkers and exploratory subgroups were examined without formal adjustment for multiple comparisons; nominal *p*-values, particularly in treatment-stratified analyses, should therefore be interpreted cautiously. Third, biomarkers were measured at a single pretreatment time point and may not reflect dynamic inflammatory changes during therapy. Fourth, treatment practice evolved over the 2017–2025 study period, particularly through increasing use of TNT. Although TNT distribution was comparable across CAP groups and adjusted sensitivity analyses incorporated cT category and TNT use, residual confounding by stage, treatment selection, and unmeasured clinical factors cannot be excluded. Fifth, ROC cut-offs were derived and evaluated in the same cohort without internal or external validation, creating a risk of optimistic performance estimates.

Other potentially relevant biomarkers, including the systemic immune-inflammation index and prognostic nutritional index, were unavailable. Survival outcomes, tumor-infiltrating lymphocyte density, and molecular characteristics such as RAS/RAF status and microsatellite instability were also not consistently available. The findings should therefore be considered exploratory and hypothesis-generating and require validation in larger prospective multicenter cohorts.

## 5. Conclusions

In this single-center retrospective cohort, selected pretreatment inflammatory biomarkers showed biologically plausible numerical patterns across CAP tumor regression groups; however, none differed significantly among the groups or demonstrated robust independent predictive performance. Monocyte count and CEA showed modest discriminatory ability for a pathological complete response, whereas neutrophil count yielded the smallest omnibus *p*-value across response groups. More favorable tumor regression was accompanied by a lower residual tumor burden and reduced nodal involvement. Nevertheless, the evaluated biomarkers should not be considered stand-alone clinical prediction tools. Larger prospective multicenter studies integrating clinical, radiological, pathological, and molecular variables are required to determine whether these readily available markers can contribute to multimodal response-prediction models.

## Figures and Tables

**Table 1:** Baseline demographic and clinicopathological characteristics according to CAP tumor regression groups.

Variable	Score 0 (*n* = 22)	Score 2 (*n* = 50)	Score 3 (*n* = 16)	*p*-Value
**Age, years (mean ± SD)**	60.8 ± 9.3	59.6 ± 10.7	61.6 ± 12.5	0.719
**Sex, M/F**	12/10	34/16	12/4	0.289
**BMI (kg/m^2^)**	25.9 ± 3.3	26.7 ± 3.2	25.8 ± 5.3	0.305
**Tumor distance from AV, cm (median [IQR])**	5 [3–7]	7 [5–9]	8 [5–10]	0.040
**LAR, n (%)**	17 (77.3%)	42 (84.0%)	11 (68.8%)	0.392
**APR, n (%)**	5 (22.7%)	8 (16.0%)	5 (31.3%)	
**ASA 2, n (%)**	6 (27.3%)	28 (56.0%)	8 (50.0%)	0.186
**ASA 3–4, n (%)**	16 (72.7%)	22 (44.0%)	8 (50.0%)	
**Length of stay, days (mean ± SD)**	7.8 ± 3.6	8.8 ± 5.4	12.1 ± 7.3	0.101
**Pretreatment cT stage**				0.051
cT2-3, n (%)	10 (45.5%)	32 (64.0%)	5 (31.3%)	
cT4, n (%)	12 (54.5%)	18 (36.0%)	11 (68.8%)	
**Pretreatment cN stage**				0.461
cN0, n (%)	7 (31.8%)	7 (14.0%)	3 (18.8%)	
cN1, n (%)	10 (45.5%)	27 (54.0%)	7 (43.8%)	
cN2, n (%)	5 (22.7%)	16 (32.0%)	6 (37.5%)	
**TNT, n (%)**	12 (54.5%)	27 (54.0%)	10 (62.5%)	0.831

BMI, body mass index; AV, anal verge; TNT, total neoadjuvant therapy; LAR, low anterior resection; APR, abdominoperineal resection; ASA, American Society of Anesthesiologists physical status classification; IQR, interquartile range; CAP, College of American Pathologists; pCR, pathological complete response. Continuous variables are presented as mean ± standard deviation (SD) or median [interquartile range (IQR)] according to data distribution and were compared using the Kruskal–Wallis test. Categorical variables are presented as numbers (percentages) and were compared using the chi-square test or Fisher’s exact test, as appropriate. Statistical significance was defined as a two-sided *p*-value < 0.05.

**Table 2:** Pretreatment pan-immune-inflammatory markers by CAP tumor regression group.

Variable	Score 0 (*n* = 22) Median [IQR]	Score 2 (*n* = 50) Median [IQR]	Score 3 (*n* = 16) Median [IQR]	*p*-Value *
**CRP (mg/L)**	6.00 [4.01–7.59]	4.39 [2.33–10.26]	4.28 [3.27–6.29]	0.877
**CEA (ng/mL)**	2.23 [1.56–4.63]	3.17 [2.09–10.54]	4.53 [2.36–18.64]	0.088
**Neutrophil (×10^3^/mm^3^)**	4.28 [3.25–5.69]	4.04 [3.37–5.13]	4.96 [4.28–6.59]	0.052
**Platelet (×10^3^/mm^3^)**	256.00 [214.00–307.00]	260.00 [220.50–329.00]	227.00 [193.00–272.25]	0.118
**Monocyte (×10^3^/mm^3^)**	0.44 [0.32–0.56]	0.55 [0.42–0.68]	0.55 [0.46–0.65]	0.075
**Lymphocyte (×10^3^/mm^3^)**	1.91 [1.32–2.69]	1.98 [1.69–2.64]	1.75 [1.49–2.42]	0.685
**NLR**	2.23 [1.64–3.08]	2.06 [1.35–2.80]	3.03 [1.98–4.06]	0.133
**PLR**	115.43 [99.92–199.40]	140.99 [101.99–192.88]	127.39 [95.43–156.29]	0.692
**PIV**	265.86 [132.42–433.92]	275.33 [196.43–489.49]	323.98 [214.95–573.78]	0.551

CRP, C-reactive protein; CEA, carcinoembryonic antigen; NLR, neutrophil-to-lymphocyte ratio; PLR, platelet-to-lymphocyte ratio; PIV, pan-immune-inflammation value; IQR, interquartile range; CAP, College of American Pathologists; pCR, pathological complete response. Continuous variables are presented as medians [interquartile range (IQR)] and were compared using the Kruskal–Wallis H test. * A two-sided *p*-value < 0.05 was considered statistically significant. Pretreatment neutrophil count, monocyte count, and CEA levels demonstrated trends toward association with tumor regression status, whereas CRP, lymphocyte count, platelet count, NLR, PLR, and PIV showed no significant differences among CAP response groups. Reference ranges: CRP 0–5 mg/L; CEA 0–5 ng/mL; neutrophil count 2–7 × 10^3^/mm^3^; lymphocyte count 0.8–4.0 × 10^3^/mm^3^; monocyte count 0.12–1.20 × 10^3^/mm^3^; platelet count 100–400 × 10^3^/mm^3^.

**Table 3:** ROC analysis and logistic regression for prediction of pathological complete response (pCR).

Variable	AUC (95% CI)	Optimal Cut-Off	Sensitivity	Specificity	Univariate OR per 1-SD Increase	95% CI	*p*-Value
**CRP**	0.535 (0.404–0.665)	≥4.000	0.773	0.439	0.482	0.140–1.653	0.246
**CEA**	0.640 (0.496–0.770)	≤2.470	0.636	0.636	0.381	0.072–2.020	0.257
**Neutrophil**	0.513 (0.370–0.654)	≥4.120	0.636	0.470	0.926	0.559–1.536	0.767
**Lymphocyte**	0.518 (0.358–0.683)	≤1.390	0.364	0.833	1.011	0.624–1.638	0.965
**Monocyte**	0.663 (0.516–0.790)	≤0.540	0.727	0.545	0.561	0.290–1.083	0.085
**Platelet**	0.501 (0.347–0.649)	≤177.000	0.182	0.924	0.997	0.616–1.616	0.991
**NLR**	0.502 (0.358–0.649)	≥1.498	0.818	0.258	1.184	0.760–1.844	0.456
**PLR**	0.521 (0.365–0.675)	≤117.935	0.545	0.652	1.201	0.765–1.886	0.427
**PIV**	0.569 (0.416–0.719)	≤138.169	0.318	0.924	0.932	0.555–1.567	0.792

CRP: C-reactive protein; CEA: carcinoembryonic antigen; NLR: neutrophil-to-lymphocyte ratio; PLR: platelet-to-lymphocyte ratio; PIV: pan-immune-inflammation value; AUC: area under the curve; CI: confidence interval; OR: odds ratio.

## Data Availability

The data presented in this study are available from the corresponding author upon reasonable request. The data are not publicly available due to institutional regulations and patient confidentiality requirements.

## Supplementary Materials

The following supporting information can be downloaded at: https://www.mdpi.com/article/10.3390/jcm15135039/s1, Table S1. Posttreatment Pathological TNM Staging According to CAP Tumor Regression Group; Table S2. Lymph-Node Parameters According to CAP Tumor Regression Group; Table S3. Covariate-Adjusted Sensitivity Analyses for Pathological Complete Response; Table S4. Exploratory Biomarker Comparisons Across CAP Groups Stratified by Treatment Type.

## Abbreviations

AJCC	American Joint Committee on Cancer
APR	Abdominoperineal Resection
ASA	American Society of Anesthesiologists
AUC	Area Under the Curve
AV	Anal Verge
BMI	Body Mass Index
CAP	College of American Pathologists
CEA	Carcinoembryonic Antigen
CI	Confidence Interval
CRC	Colorectal Cancer
CRP	C-Reactive Protein
CRT	Chemoradiotherapy
dMMR	Deficient Mismatch Repair
IQR	Interquartile Range
LARC	Locally Advanced Rectal Cancer
LAR	Low Anterior Resection
MRI	Magnetic Resonance Imaging
MSI-H	Microsatellite Instability-High
nCRT	Neoadjuvant Chemoradiotherapy
NLR	Neutrophil-to-Lymphocyte Ratio
OR	Odds Ratio
pCR	Pathological Complete Response
PIV	Pan-Immune-Inflammation Value
PLR	Platelet-to-Lymphocyte Ratio
ROC	Receiver Operating Characteristic
SCRT	Short-Course Radiotherapy
SD	Standard Deviation
TAM	Tumor-Associated Macrophage
TME	Total Mesorectal Excision
TNT	Total Neoadjuvant Therapy
TRG	Tumor Regression Grade
