# Supplementary material for: Association of Pretreatment Immune-Inflammatory Biomarkers with Pathological Tumor Regression Following Neoadjuvant Chemoradiotherapy in Locally Advanced Rectal Cancer
Source: J Clin Med. 2026 Jun 28;15(13):5039. doi: 10.3390/jcm15135039 (PMC13362307; doi:10.3390/jcm15135039)
Supplement: Supplementary file 1 [file jcm-15-05039-s001.zip › jcm-4380588-supplementary.pdf]

Supplementary Materials

Association of Pretreatment Immune-Inflammatory Biomarkers With Pathological Tumor Regression Following Neoadjuvant Chemoradiotherapy in Locally Advanced Rectal Cancer

Supplementary Table S1. Posttreatment Pathological TNM Staging According to CAP Tumor Regression Group

Stage variable	CAP 3 (n=16)	CAP 2 (n=50)	CAP 0 (n=22)	p-value
Posttreatment ypT stage				<0.001
ypT0, n (%)	0 (0.0%)	0 (0.0%)	22 (100.0%)	
ypT1, n (%)	0 (0.0%)	2 (4.0%)	0 (0.0%)	
ypT2, n (%)	2 (12.5%)	22 (44.0%)	0 (0.0%)	
ypT3, n (%)	9 (56.2%)	23 (46.0%)	0 (0.0%)	
ypT4A, n (%)	2 (12.5%)	2 (4.0%)	0 (0.0%)	
ypT4B, n (%)	3 (18.8%)	1 (2.0%)	0 (0.0%)	
Posttreatment ypN stage				<0.001
ypN0, n (%)	4 (25.0%)	37 (74.0%)	22 (100.0%)	
ypN1A, n (%)	1 (6.2%)	3 (6.0%)	0 (0.0%)	
ypN1B, n (%)	6 (37.5%)	1 (2.0%)	0 (0.0%)	
ypN1C, n (%)	2 (12.5%)	8 (16.0%)	0 (0.0%)	
ypN2/2A/2B, n (%)	2 (12.5%)	1 (2.0%)	0 (0.0%)	

Chi-square test or Fisher's exact test, as appropriate. CAP, College of American Pathologists.

Supplementary Table S2. Lymph-Node Parameters According to CAP Tumor Regression Group

Parameter	CAP 3 (n=16)	CAP 2 (n=50)	CAP 0 (n=22)	p-value
LN examined, mean ± SD	18.44 ± 8.30	15.38 ± 7.62	11.45 ± 4.21	0.006
LN examined, median [IQR]	16.5 [12.75–19.00]	14.5 [10.25–18.00]	12.0 [7.25–13.00]	
Positive LN, mean ± SD	3.00 ± 5.15	0.34 ± 1.12	0.00 ± 0.00	<0.001
Positive LN, median [IQR]	2.0 [0.00–4.00]	0.0 [0.00–0.00]	0.0 [0.00–0.00]	
Any nodal disease (ypN+), n (%)	12 (75.0%)	13 (26.0%)	0 (0.0%)	<0.001
ypN0, n (%)	4 (25.0%)	37 (74.0%)	22 (100.0%)	<0.001

Kruskal–Wallis tests were used for continuous variables; LN, lymph node; IQR, interquartile range; SD, standard deviation.

Supplementary Table S3. Covariate-Adjusted Sensitivity Analyses for Pathological Complete Response

Model	Variable	Adjusted OR	95% CI	p-value
Model 1	CEA, per 1-SD increase	0.357	0.066–1.932	0.232
	cT4 vs. cT2–3	1.750	0.651–4.703	0.267
	TNT, yes vs. no	0.968	0.359–2.608	0.948
Model 2	Neutrophil count, per 1-SD increase	0.899	0.541–1.494	0.681
	cT4 vs. cT2–3	1.571	0.591–4.173	0.365
	TNT, yes vs. no	0.919	0.345–2.444	0.865
Model 3	Monocyte count, per 1-SD increase	0.547	0.276–1.082	0.083
	cT4 vs. cT2–3	1.404	0.518–3.805	0.505
	TNT, yes vs. no	0.726	0.260–2.026	0.541

pCR (ypT0N0) was coded as the positive outcome. Each model included one standardized biomarker and was adjusted for baseline cT category and TNT use. Biomarker ORs are reported per one-standard-deviation increase. CEA, carcinoembryonic antigen; CI, confidence interval; OR, odds ratio; TNT, total neoadjuvant therapy. Total n=88; pCR events=22.

Supplementary Table S4. Exploratory Biomarker Comparisons Across CAP Groups Stratified by Treatment Type

Treatment subgroup	Biomarker	CAP 3 median [IQR]	CAP 2 median [IQR]	CAP 0 median [IQR]	p-value
Conventional long-course nCRT	CRP	5.580 [4.600–5.862]	5.030 [2.835–16.340]	6.000 [4.075–7.245]	0.914
	CEA	10.650 [2.843–22.815]	3.610 [2.095–6.640]	2.095 [1.558–4.177]	0.209
	Neutrophil	4.735 [3.025–6.258]	4.100 [3.455–5.185]	4.630 [2.772–6.173]	0.923
	Platelet	220.000 [194.500–256.750]	316.000 [241.500–356.000]	272.000 [250.750–291.000]	0.107
	Monocyte	0.695 [0.520–0.848]	0.630 [0.475–0.770]	0.475 [0.430–0.578]	0.205
	Lymphocyte	1.900 [1.578–2.058]	1.900 [1.635–2.355]	1.475 [1.143–2.360]	0.848
	NLR	3.422 [2.259–3.695]	2.343 [1.538–3.633]	2.492 [1.953–3.616]	0.810
	PLR	132.760 [127.181–143.652]	177.215 [132.186–199.008]	166.311 [115.588–241.005]	0.527
	PIV	443.688 [199.335–676.356]	428.936 [227.921–719.614]	355.742 [231.002–600.309]	0.832
TNT	CRP	3.350 [2.598–6.383]	4.120 [2.010–7.585]	5.725 [3.717–8.280]	0.676
	CEA	3.680 [2.535–12.400]	3.150 [2.095–14.850]	2.405 [1.665–5.790]	0.410
	Neutrophil	4.965 [4.920–6.535]	3.770 [3.355–4.730]	4.280 [4.013–4.850]	0.010
	Platelet	227.000 [197.000–280.750]	257.000 [213.000–291.500]	232.500 [186.250–319.500]	0.746
	Monocyte	0.510 [0.448–0.565]	0.510 [0.380–0.610]	0.400 [0.287–0.538]	0.209
	Lymphocyte	1.725 [1.467–2.473]	2.110 [1.700–2.810]	2.035 [1.667–2.797]	0.550
	NLR	2.737 [2.014–4.200]	1.856 [1.284–2.513]	1.832 [1.435–2.878]	0.081
	PLR	121.019 [94.477–172.423]	123.837 [90.235–158.630]	109.785 [91.801–129.523]	0.821
	PIV	320.107 [217.402–461.124]	228.033 [172.621–369.331]	164.313 [99.848–400.421]	0.273

Group sizes were CAP 3/CAP 2/CAP 0 = 6/23/10 for conventional nCRT and 10/27/12 for TNT. Kruskal–Wallis tests were used. These analyses were exploratory, involved multiple comparisons, and were not adjusted for multiplicity; nominal p-values should therefore be interpreted cautiously. nCRT, neoadjuvant chemoradiotherapy; TNT, total neoadjuvant therapy.
